# Immune oncology, immune responsiveness and the theory of everything

**DOI:** 10.1186/s40425-018-0355-5

**Published:** 2018-06-05

**Authors:** Tolga Turan, Deepti Kannan, Maulik Patel, J. Matthew Barnes, Sonia G. Tanlimco, Rongze Lu, Kyle Halliwill, Sarah Kongpachith, Douglas E. Kline, Wouter Hendrickx, Alessandra Cesano, Lisa H. Butterfield, Howard L. Kaufman, Thomas J. Hudson, Davide Bedognetti, Francesco Marincola, Josue Samayoa

**Affiliations:** 1 Immune-Oncology Discovery, AbbVie, Redwood City, CA USA; 2 Clinical Pharmacology and Pharmacometrics, Abbvie, Redwood City, CA USA; 3 Immune-Oncology, AbbVie, North Chicago, IL USA; 4 Tumor Biology, Immunology, and Therapy Section, Division of Translational Medicine, Research Branch, Sidra Medical and Research Center, Doha, Qatar; 5 Nanostring Technologies, Inc., Seattle, WA USA; 6 UPMC Hillman Cancer Center, University of Pittsburgh, Pittsburgh, PA USA; 7 Departments of Surgery and Medicine, Rutgers University, New Jersey, NJ USA

**Keywords:** Cancer immunotherapy, Checkpoint inhibitors, Immune resistance

## Abstract

Anti-cancer immunotherapy is encountering its own checkpoint. Responses are dramatic and long lasting but occur in a subset of tumors and are largely dependent upon the pre-existing immune contexture of individual cancers. Available data suggest that three landscapes best define the cancer microenvironment: ***immune-active***, ***immune-deserted and immune-excluded***. This trichotomy is observable across most solid tumors (although the frequency of each landscape varies depending on tumor tissue of origin) and is associated with cancer prognosis and response to checkpoint inhibitor therapy (**CIT**). Various gene signatures (e.g. Immunological Constant of Rejection - **ICR** and Tumor Inflammation Signature **- TIS**) that delineate these landscapes have been described by different groups. In an effort to explain the mechanisms of cancer immune responsiveness or resistance to CIT, several models have been proposed that are loosely associated with the three landscapes. Here, we propose a strategy to integrate compelling data from various paradigms into a “*Theory of Everything*”. Founded upon this unified theory, we also propose the creation of a task force led by the Society for Immunotherapy of Cancer (***SITC***) aimed at systematically addressing salient questions relevant to cancer immune responsiveness and immune evasion. This multidisciplinary effort will encompass aspects of genetics, tumor cell biology, and immunology that are pertinent to the understanding of this multifaceted problem.

**Electronic supplementary material:**

The online version of this article (10.1186/s40425-018-0355-5) contains supplementary material, which is available to authorized users.

## Premise and background

Anti-cancer immunotherapy is encountering its own checkpoint. Responses are dramatic and long lasting but occur in a subset of tumors and are largely dependent upon the pre-existing immune contexture of individual cancers [1]. Current research is trying to determine why some cancers respond to CIT more than others and the reasons for individuals’ variability within each indication [2, 3].

Several morphological observations based on immune histochemical analyses suggest that three immune landscapes best define distinct varieties of the cancer microenvironment: an ***immune-active***, an opposite ***immune-deserted*** and an intermediate ***immune-excluded*** [4]*.* Across cancers, and among subtypes, the prevalence of each landscape may differ. Nevertheless, this trichotomy is observable across most solid tumors suggesting that convergent evolutionary adaptations determine the survival and growth of cancer in the immune competent host leading to predictable patterns determined by uniform immunological principles independent of the biology pertinent to distinct tumor tissue of origin. It is therefore reasonable to postulate that the mechanisms leading to cancer resistance to checkpoint blockade are similar across cancers deriving from different tissues. Functional characterization based on transcriptional analyses cannot distinguish structural differences. Thus a reductionist argument could be made that at the functional level cancers can simply be aggregated into ***immune-active*** or ***immune-silent clusters.*** Current work from our group suggests that most immune excluded cancer resemble functionally immune active tumors suggesting that the periphery immune cells interact with cancer cells (unpublished observation).

We will refer to the mechanisms allowing persistence of cancer in the immune-active cluster as ***Compensatory Immune Resistance*** (***CIRes***) based on the assumption that lack of CIRes would prevent tumor survival against the host’s immune response. Conversely, we refer to survival of cancer in the immune-deserted environment as ***Primary Immune Resistance*** (***PIRes***). In 2002, we proposed that human cancer immune responsiveness to antigen-specific vaccination administered in combination with systemic interleukin-2 is predetermined by a tumor microenvironment conducive to immune recognition [5] Likewise, recent observations suggest that CIT is most effective for the treatment of immune active tumors, where a tenuous balance between immune-effector and immune-suppressive mechanisms determines outcomes [6–8].

To explain CIRes and PIRes, several phenomenologies have been described and models proposed that largely outnumber the fewer immune landscapes (Table 1). Such discrepancy can be explained in three ways: a) some models do not translate broadly across the majority of human cancers, b) there are subtler immune landscapes than those discernable by current approaches, or c) some models are redundant and describe different facets of the same pathophysiology. To solve this discrepancy, we surveyed human cancers through readily-available open-access information.

**Table 1:** Principal models related to immune responsiveness

	Immune Landscape^a^	References
WNT/βCatenin	Silent (0.03)	[38, 39]
MAPK Hypothesis	Silent (0.001)	[10]
Immunogenic Cell Death	Active (< 0.001)	[19], [20, 21]
Regulatory T cells	Active (< 0.001)	[24, 25]
IL23-Th17 Axis	Active (< 0.001)	[26, 41–44]
Myeloid Suppressor Cells	Active (< 0.001)	[50]
PI3K-γ Signature	Active (< 0.01)	[52–55, 63]
IDO/NOS Signature	Active (< 0.01)	[51, 81, 82]
SGK1 Signature	Ubiquitous	[56, 57]
Shc1 signature	Ubiquitous	[62]
Barrier Molecules	Ubiquitous	[27, 28]
Mesenchymal Transition	Ubiquitous	[29, 30, 83]
Cancer-Associated Fibroblasts	Ubiquitous	[31–35, 84]
TAM receptor tyrosine kinases	Ubiquitous	[47, 58–60, 85]
Hypoxia/Adenosine suppression	Ubiquitous	[48, 49]
TREX1clearence of Cytosolic DNA	NA	[86, 87]
Checkpoint Cluster	Active (< 0.001)	[22, 23]
oncogene addicted tumors	Silent	[11, 68]
Epigenetic Regulation	Ubiquitous	[12, 88–90]

^a^Distinct models have been assigned to either the Silent or the Active Landscape according to the results of the survey shown in Fig. 1**.** Ubiquitous refers to models that are not significantly associated with either immune landscape

Marincola et al. [9] have previously described a transcriptional signature comprising the concordant activation of innate and adaptive immune effector mechanisms that is required for the occurrence of ***immune-mediated tissue-specific destruction***. This represents a conserved mechanism determining destructive autoimmunity, clearance of pathogen-bearing cells during acute infection, acute allograft rejection, graft-versus-host disease and rejection of cancer. Thus, the signature was termed: the ***Immunologic Constant of Rejection (ICR)*** [9]. The ICR signature was derived from bulk tumor transcriptome data sets, as they offer the most readily-available sample/data type and the easiest to apply in the clinic due to the ease of collection. The ICR signature was further trained to be representative of the broader signature as previously described [10] and is currently represented by twenty transcripts and four functional categories: CXCR3/CCR5 chemokines (including *CXCL9*, *CXCL10*, *CCL5*), Th1 signaling (including *IFNG*, *IL12B*, *TBX21*, *CD8A*, *STAT1*, *IRF1*, *CD8B*), effector (including *GNLY*, *PRF1*, *GZMA*, *GZMB*, *GZMH*) and immune regulatory (including *CD274*, *CTLA4*, *FOXP3*, *IDO1*, *PDCD1*) functions. The expression of these twenty representative genes is highly correlated with the extended ICR signature that includes approximately five-hundred transcripts and is representative of its main functional orientation as previously described [11, 12]. Importantly, the specific cell types in the tumor microenvironment expressing these genes will ultimately be relevant in elucidating the mechanistic link between the ICR and the immune responsiveness of cancer. It was subsequently observed that the ICR serves both as a positive predictor of responsiveness to immunotherapy and as a favorable prognostic marker for various tumor types [6, 10, 13, 14]. This observation suggests that these related phenomena represent facets of a spectrum within the continuum of anti-cancer immune surveillance. Such continuity leads to the fair, though unproven, assumption that signatures predictive of prolonged survival may mark an immune-favorable cancer phenotype and serve as surrogate predictors of responsiveness to anti-cancer immunotherapies [10, 15]. This assumption is also corroborated by recent reports suggesting that similar gene expression patterns predict response to CIT [6–8]. Specifically, Ayers et al. [6] using RNA from pre-treatment tumor samples of pembrolizumab-treated patients and the nCounter platform identified and validated a pan-tumor T-cell–inflamed gene signature correlating with clinical benefit. This ***tumor inflammation signature*** (***TIS***) contains IFN-γ–responsive genes (*CD27*, *STAT1*, *IDO1*, *HLA-E*, *NKG7*) related to antigen presentation (*HLA-DQA1*, *HLA-DRB1*, *PSMB10*, *CMKLR1*) chemokine expression (*CCL5*, *CXCL9*, *CXCR6*), cytotoxic activity (*CD8A*), and adaptive immune resistance (*TIGIT*, *LAG3*, *CD274*, *CD276*, *PDCD1LG2*) and as such, is highly correlated to the ICR signature: composite scores for each signature calculated with ssGSEA software and compared according the expression values in the 999 breast cancer samples from TCGA were highly correlated (*r* = 0.98). The TIS has been developed into a clinical grade assay running on the nCounter platform currently being evaluated in ongoing pembrolizumab trials (3). We, therefore, developed a strategy to build a **navigational map of cancer immunity** with the primary purpose of assigning distinct immune responsive and resistant models to their respective immune-landscapes using the expression of twenty transcripts that are representative of the extended ICR signature.

Using the ICR signature [10], we queried the prognostic accuracy of a transcriptional data set of breast cancers from ***The Cancer Genome Atlas*** (***TCGA***) as a discovery platform and validated the findings on a second transcriptional set of breast cancers from the ***Gene Expression Omnibus*** (***GEO***) repository at the National Center for Biotechnology Information. The TCGA set encompasses RNA-seq-based transcriptional characterization of 999 breast cancer cases while the compilation of 10 GEO studies included 1728 cases of breast cancer (compiled in [16]) that were transcriptionally characterized utilizing a uniform Affymetrix platform. Both datasets were classified according to the coordinated expression of ICR transcripts [10].

**Fig. 1:**
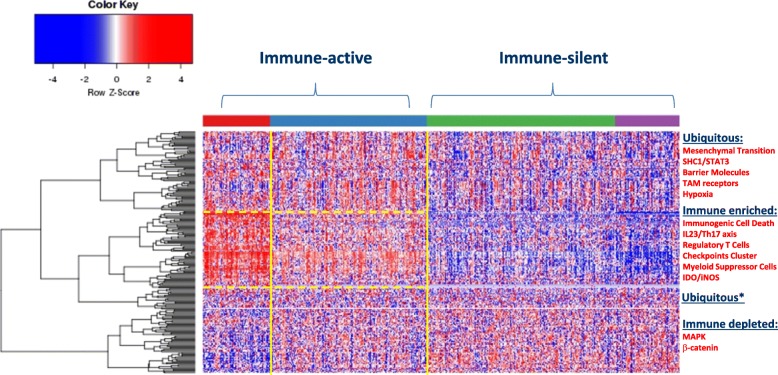
Distribution of sRes gene expression according to distinct models (Table 1) within immune landscapes as defined by ICR gene expression. Four immune landscapes were identified ranked according to the level of expression of ICR genes with purple, green, blue and red representing respectively ICR 1, 2, 3 and 4. Because of similarities in patterns of gene expression, for the purpose of discussion the landscapes will be referred to as immune-silent (ICR1–2) or Immune-active (ICR3–4). Genes were assigned to distinct landscapes according to significant difference in expression between ICR4 and ICR1 (*p*-value < 0.05 and false discovery rate < 0.1). Genes signatures associated with a particular immune responsiveness model as per Table 1 were assigned to distinct landscapes according to gene enrichment analysis and ubiquitous were considered signatures that did not reach significance (one-tailed Fisher Test p-value < 0.01). *Cluster of ubiquitous genes that segregate with the immune active signatures but did not reach significance and, therefore, were considered ubiquitous

ICR groups were ranked 1–4, according to the level of expression of the 20 representative ICR genes (Fig. 1). At the transcriptional level a dichotomy between Immune-active (ICR3–4) and immune-silent (ICR1–2) clusters was apparent [10]. Kaplan-Meier applied to the four ICR classes confirmed that ICR gene expression correlates with survival in breast cancer [10].

Subsequently, we collected transcriptional ***Signatures of Responsiveness*** (or Resistance) (***sRes***) as reported by other investigators (Table 1) and assessed them for their distribution within the four ICR groups (Fig. 1). The signatures tested and respective publication from which the transcript biomarkers were derived are available in Table 1. We recognize that the current collection of sRes is far from being comprehensive nor reflective of all proposed models of immune resistance and/or responsiveness. While further work is being entertained to refine and update the collection according to novel understanding of cancer immune biology, for the purpose of this commentary the current version sufficiently highlights the process that we are proposing.

Self-organizing clustering of sRes signatures demonstrated a preferential distribution of immune suppressor activities such as those related to Th17-IL23 axis, T regulatory cells, checkpoint cluster, myeloid suppressor cells, IDO within the ICR4 and, to a lesser degree, the ICR3 immune landscapes (Fig. 1). This finding defines an immune phenotype of breast cancer enriched in concert with immune effector and immune suppressive mechanisms. Not surprisingly, the transcriptional signature representative of immunogenic cell death was included in the immune active landscape. This information presents a strong argument for the existence of CIRes mechanisms balancing immune pressure in these cancers’ evolutionary processes.

Conversely, the immune depleted landscapes (ICR1 and ICR2) belonging to the immune silent cluster were best explained by PIRes, lacking evidence for the priming of a genuine immune response. The sRes of this cluster is enriched with transcripts in the PI3Kγ/SFK/pGSK3/β-catenin axis, and activation of the signal transducer and activator of transcription (**STAT3**). Coincidentally, these sRes are also associated with suppressive myeloid cell differentiation and activation of the IL-23/Th17 axis. However, activation of the PI3Kγ/SFK/pGSK3/β-catenin axis does not correspond to activation of immunologic transcripts within the same cluster.

In conclusion, this survey suggested that:

1. In immune active tumors, signatures of immune suppression and activation are both present and this balance is responsible for CIRes in the ICR4, and to a lesser degree the ICR3, subclasses of breast cancer.
2. Immune active tumors (ICR3–4) are enriched in sRes and immunogenic signatures enriched for:
  - Immunogenic Cell Death activation
  - IL23/Th17,
  - Checkpoints cluster
  - Myeloid suppressor cells
  - Regulatory T cells
  - IDO
3. Immune-silent tumors are enriched with signatures reflecting activation of STAT3 and the PI3Kγ/SFK/pGSK3/β-catenin axis and their depletion of immune regulatory mechanisms argues for PIRes:
  - β-catenin
  - MAPK activation

Thus, the various models of immune resistance (Table 1) converge either into PIRes or CIRes. Interestingly, the CIRes signatures are co-expressed with those reflecting STING activation [17, 18] and immunogenic cell death [19–21]. This observation suggests that immunogenicity must be balanced by immune suppression in immune active tumors.

In an effort to move these in silico observations toward clinical validation and novel biology-based strategies of immune-modulation, new molecular tools which can be reproducibly applied in the clinic are needed. A possible candidate is the PanCancer IO 360 Gene Expression Panel (Nanostring), which allows for multi-plexed targeted exploration of genes involved in the tumor-immune microenvironment, allowing for a multifaceted characterization of disease biology and interrogation of mechanisms of immune evasion. This panel was developed specifically for translational research and incorporates many of the PIRs and CIRes signatures including the ICR and the TIS.

## Discussion

Several models have been proposed to explain proclivity or resistance of cancer in response to immunotherapy (Table 1). Effector T cell exhaustion is broadly observed in the tumor microenvironment manifesting through the expression of a cluster of immune checkpoints often concomitantly expressed in response to chronic interferon stimulation [22, 23]. In addition, it is well established that regulatory T cells balance immune effector mechanisms [24–26]. Other models propose blockade of immune cell homing to cancer tissue by barrier molecules, chemo-inhibitory mechanisms, and by epigenetic silencing of chemokines (*CCL5*, *CXCL9*, and *CXCL10*), Th1 signaling molecules and antigen processing machinery components [12, 27–37].

Other immune resistance models point to alterations of cancer cell signaling that result in secondary dysregulation of myeloid cell function. Cancer-intrinsic β-catenin signaling defects disrupt chemo-attraction of dendritic cells (**DCs**) and, consequently, antigen presentation in the context of immunogenic cell death [21, 38–40]. In addition, polarization of DCs toward a tolerogenic, IL23 producing phenotype leading to Th17 polarization was described in experimental animal models and in human samples [26, 41–46]. Suppression of anti-cancer immunity has also been attributed to the TAM receptor tyrosine kinase family members that mediate efferocytosis and negative regulation of DC activity [47]. Similarly, hypoxia can drive immune suppression by inducing tolerogenic myeloid DC polarization [48, 49]. Finally, myeloid cell biology is responsible for the immune regulation of the cancer microenvironment through the upregulation of metabolizing enzymes such as arginase and indoleamine 2,3-dioxygenase, which can negatively impact T cell function [50, 51].

**Fig. 2:**
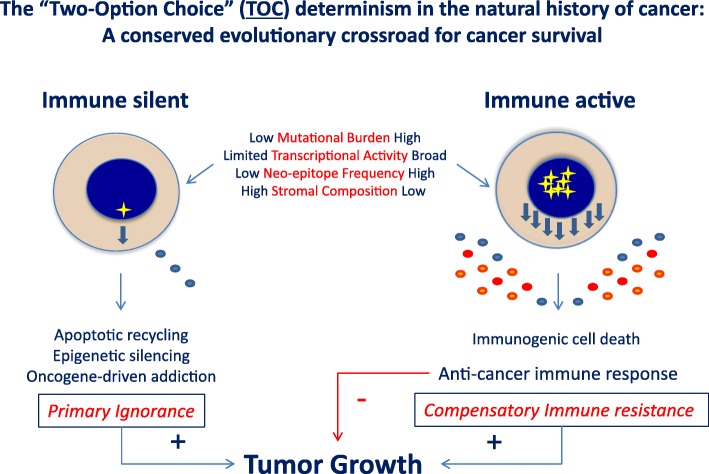
Dichotomy in the Myeloid-Centric Hypothesis of immune resistance: the same pathway is relevant to myeloid cell differentiation as well as intrinsic oncogenic activation (in red boxes are included models included in Table 1). It is currently unclear how the two interpretations diverge vs relate to each other and further characterization of the single cell level will need to be entertained to clarify this point

The phenotype of suppressive myeloid cells in the microenvironment is often attributed to activation of the PI3Kγ/SFK/pGSK3/β-catenin axis (Fig. 2). Phosphoinositide3-kinase-gamma (**PI3Kγ**) can act as a molecular switch that triggers immune suppressive mechanisms in myeloid DCs [52, 53]. At the same time, alteration of PI3K functional components plays a widespread role in tumorigenesis [54]. Downstream phosphorylation of serum and glucocorticoid kinase 1 (**SGK1**) by the PI3K/PDK1 cascade leads to activation of glycogen synthase kinase 3 beta (**GSK3β**) and subsequently β-catenin [55–57]. Interestingly, most studies describing dysregulation of the PI3Kγ/SFK/pGSK3/β-catenin axis refer to abnormalities intrinsic to tumor cells, although the same pathway can play an important role in myeloid suppressor DC induction and immune suppression downstream of the TAM receptor tyrosine kinases [58–60]. Converging on the same pathway, hypoxia inducible factors (HIF1α) signal through the SGK3β/β-catenin axis promoting cancer cell stemness and immune suppression [48, 49, 61] (Fig. 2).

An upstream inducer of PI3Kγ stimulation is the scaffold protein SHC1 that shifts the balance between STAT1 and STAT3 activation in favor of the latter, promoting immune suppression [62]. The context in which activation of SHC1 preferentially regulates myeloid DC polarization versus cancer cell signaling remains unclear. Similarly, loss of protein tyrosine phosphatase non-receptor type 2 (**PTPN2**) function that inhibits PI3Kγ signaling is associated with activation of the tumorigenic pathway, while at the same time can modulate T cell function through mDC activation [63, 64] and induction of Th17 polarization [65, 66]. Finally activation of the mitogen-activated protein kinases (**MAPKs**) programs is consistently observed in immune silent tumors and is associated with a respective mutational signature [10].

Therefore, it may be that most models of immune resistance are based on a diverse interpretation of the disruption of the PI3Kγ/SFK/pGSK3/β-catenin pathway: one centered on tumorigenesis and the other on myeloid cell biology however it is currently unclear whether the two mechanisms are mutually exclusive or can be observed in association in the immune active tumors. This question can only be solved by morphological documentation of cell-specific activation of the pathway either by immunohistochemistry or by single cell transcriptional analysis. However, according to our results and the published literature [10, 38, 67], it appears that the former interpretation pertains most prominently to the immune silent cluster (PIRes) while the latter appears to be most likely pertaining to the immune active (CIRes, Fig. 2).

These results may bear remarkable impact for the design of combination therapies. It appears that, at least in breast cancer, therapeutic combinations directed against immune regulatory mechanism (i.e. checkpoint blockade, IL-23/Th17, TAM receptor kinases, hypoxia factors or IDO inhibitors) will modulate and possibly enhance responsiveness of cancers with CIRes (immune active cluster) but will be unlikely to work in the context of immune silent cancers of the PIRes phenotype unless complimentary efforts are made to disrupt the non-immunogenic landscape to convert it into an immunogenic one.

**Fig. 3:**
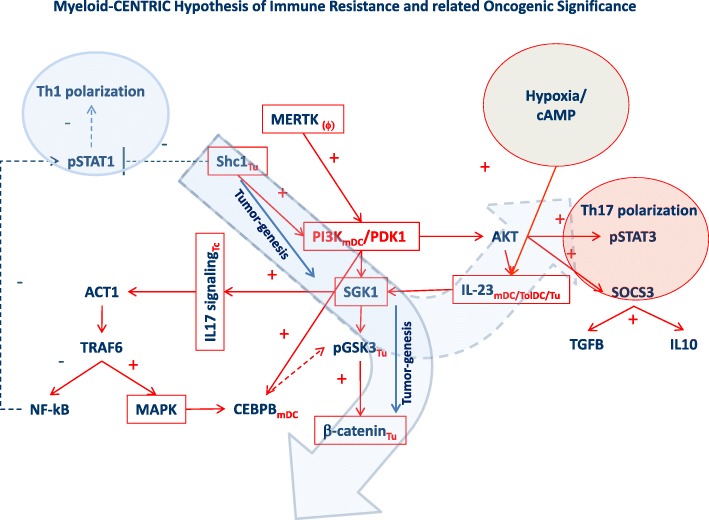
The two-option choice or Hobson’s predicament in cancer survival

We hypothesize that immune silent tumors evolve by employing a strictly essential interface of interactions with the host’s stroma that limits immune cell recognition. This may be due to the selection of a growth process devoid of immunogenic cell death (Fig. 1). Thus, these “clean” tumors evolve through the selection of cancer cells that adopt refined growth mechanisms reduced to the bare necessities of life. Indeed, preclinical and clinical data focused on molecular subtypes of clinically-validated oncogene-addicted tumors (e.g., ALK+, EGFR+, BRAFV600E+, NTRK-rearranged tumors) indicate that these tumors often portray minimal CD8+ T cell infiltration along with reduced expression of immunosuppressive factors [11, 68]. These molecular subtypes of EGFR-mutated or ALK+ non-small cell lung cancer (NSCLC) serve as a perfect clinically validated example of “clean tumors” as these tumors usually do not have high mutational burden, occur in younger patients, and in non-smokers. This is supported by recent evidence which demonstrates that presence of oncogenic driver mutations in NSCLC, such as EGFR, ALK, ROS1, RET fusions and C-MET exon 14 skipping is associated with lower mutational burden (Mohamed E. Salem, ASCO presentation 2017, http://abstracts.asco.org/199/AbstView_199_184601.html). This hypothesis is further corroborated by the observation that these tumors bear a low prevalence of mutations in oncogenes suggesting a more orderly growth process [10]. It is, therefore, reasonable to suppose that the growth of clean (“oncogene addicted”) tumors is dependent on activation of specific pathways (e.g. the PI3Kγ/SFK/pGSK3/β-catenin axis) that avoid immune recognition. Thus, we propose that the natural history of cancer is shaped at the crossroad of two biologies by a “Two-Option Choice”: 1) immunogenic tumors evolve through a disorderly trial-and-error accumulation of oncogenic processes generated by their intrinsic genetic instability that leads to a broader number of host-immune interactions. These tumors can, therefore, only survive in the immune competent host when immune suppressive mechanisms balance the immune reaction, 2) silent tumors follow a more orderly process with a sequential accumulation of essential genetic traits and can grow undisturbed by the immune system (Fig. 3). Since the latter appear to depend on a leaner carcinogenesis, it may be reasonable to postulate that disruption of this delicate survival skill may induce messier cancer biology prone to immunogenic cell death. Whether this is true remains to be tested. Turning an immune silent into immune active tumor microenvironment, even temporarily, may serve a critical therapeutic role opening the door for immunotherapy strategies. This in turn may be critical because successful anti-cancer immunotherapy induces durable tumor regression and immune memory more frequently.

In conclusion, we propose a systematic, hypothesis-driven task force led by SITC to prioritize and address the salient questions related to cancer immune responsiveness based on a deeper understanding of the cancer cell biology that orchestrates distinct immune landscapes. The task force should address outstanding questions to identify conserved versus peculiar patterns of immune interaction between the host and cancers of different ontology. The role that the genetic background of the host or micro environmental modifiers play in regulating cancer-immune biology should be addressed following appropriate stepwise approaches [69]. In addition, a deeper understanding of the evolutionary processes shaping the development of cancer in the immune competent host may offer a simplified understanding of conserved mechanisms of cancer survival and consequently help the identification of a broad range of therapeutics that can target dominant pathways leading to immunogenic cancer cell death. A clearer qualification of the role played by adaptive versus innate mechanisms in initiating immune activation should be considered. Two non-exclusive yet divergent lines of thought are raised to explain immunogenic cancer biology: on one side the high prevalence of neo-epitopes predicted by the higher mutational burden observed in immunogenic tumors positions adaptive immune recognition at the forefront of immune activation [70–74]. Conversely, immunogenic cell death may primarily drive inflammation with secondary recruitment of immune cells [20, 21, 75, 76]. The role that each mechanism plays in human cancer biology, and its implication for therapeutic intervention, remains to be clarified, and better integrated tools may improve our holistic understanding of the underlying cancer-immune biology thus facilitating novel biology-based combinational therapeutic strategies.

Finally, better in vivo (genetically engineered and/or syngeneic) rodent models for the screening of therapeutic strategies should be better characterized [77–79]. Some animal models may be reflective of immune-activated landscapes and be most relevant for the definition of therapies combining immune modulatory agents. Other animal models may more closely resemble the biology of immune-silent cancers and would be best utilized to identify therapies that can initiate an immune response before immunomodulatory agents are introduced sequentially and/or combinatorically. The availability of complimentary mouse/human paired panels would largely facilitate such efforts. To our knowledge, little has been done so far to match mouse models to corresponding human immuno-oncology phenotypes following the perspective proposed by this unified theory of everything.

The Taskforce will define its goals and future activities in the occasion of a foundational workshop to be held in San Francisco on May 14–15 2018 (SITC Cancer Immune Responsiveness Workshop).

The topics to be discussed will include:

- Interactions between tumor evolution in the immune competent host and the resulting immune landscape
- Identification of common pathways that could be interrogated and targeted to better understand and increase immunogenicity among silent or ‘cold’ cancers
- Mechanistic understanding of parameters that could predict immune response to different cancer immunotherapies
- Development of animal models that accurately reflect the immune landscape in ‘hot’ versus ‘cold’ human tumors

This workshop will be held in tandem with the SITC Biomarkers Workshop to be held subsequently on May 16–17 in the same premise as part of a strong interest by SITC and other organizations [80] to deepen the understanding of cancer immune biology particularly in association with clinical trial development: (SITC Biomarkers Workshop).

## Methods

All data download, processing and analyses were done in R programming environment and as described in Hendrickx et al. [11]. For the unsupervised clustering of the TOE genes (Additional file 1), modified distance and hierarchical clustering functions were used. Specifically the distance between 2 genes was defined as 1-“Correlation Coefficient (Spearman)” and for the hierarchical clustering function “Ward.D2” method was used.

Composite correlation between the ICR and the TIS signature was assessed by calculating a cumulative score for each gene included in the respective signature using ssGSEA method form GSVA package and correlating the scores in the breast cancer TCGA data set according to Spearman Correlation.

The metrics used when assigning genes to silent, active and ubiquitous groups are derived from differential expression statistics between ICR1 and ICR4 samples. The genes are assigned to the active cluster if they have significantly higher expression levels in ICR4 samples (*p*-value < 0.05 and FDR < 0.1). Similarly the genes are assigned to the silent cluster if they have significantly higher expression levels in ICR1 samples (p-value < 0.05 and FDR < 0.1). If the genes do not pass these cutoffs they are grouped as “Ubiquitous”. Geneset enrichment for each signature belonging to individual models of immune resistance (Table 1) against ICR1 and ICR4 clusters was assessed using one-tailed Fisher’s exact test.

In the analyses and corresponding heatmaps, the genes that were identified in multiple signatures were plotted as one, so each gene in the heatmap is unique. When the ICR direction is inferred for each signature, the repeated genes contributed to each signature with the same statistics.

## Additional file

Additional file 1: List of individual genes and corresponding models related to immune suppression used for the Theory of Everything (TOE). (TXT 11 kb)

## Abbreviations

CIRes: Compensatory Immune Resistance
DC: Dendritic Cell
GEM: Genetically Modified Mouse Models
GEO: Gene Expression Omnibus
GSK: Glycogen synthase kinase
ICR: Immunologic Constant of Rejection
IDO: Indoleamine 2,3-dioxygenase
MAPK: Mitogen-activated protein kinase
PI3K: Phosphoinositide3-kinase-gamma
PIRes: Primary Immune Resistance
PTPN2: Protein tyrosine phosphatase non-receptor type 2
SGK: Serum and glucocorticoid kinase
sRes: Signatures of Resistance
STAT: Signal transducer and activator of transcription 1
TCGA: The Cancer Genome Atlas
TCIA: The Cancer Immunome Atlas
TOC: Two-Option Choice
TOE: Theory of Everything
